# Machine learning classification of conduct disorder with high versus low levels of callous-unemotional traits based on facial emotion recognition abilities

**DOI:** 10.1007/s00787-021-01893-5

**Published:** 2021-10-18

**Authors:** Ruth Pauli, Gregor Kohls, Peter Tino, Jack C. Rogers, Sarah Baumann, Katharina Ackermann, Anka Bernhard, Anne Martinelli, Lucres Jansen, Helena Oldenhof, Karen Gonzalez-Madruga, Areti Smaragdi, Miguel Angel Gonzalez-Torres, Iñaki Kerexeta-Lizeaga, Cyril Boonmann, Linda Kersten, Aitana Bigorra, Amaia Hervas, Christina Stadler, Aranzazu Fernandez-Rivas, Arne Popma, Kerstin Konrad, Beate Herpertz-Dahlmann, Graeme Fairchild, Christine M. Freitag, Pia Rotshtein, Stephane A. De Brito

**Affiliations:** 1 Centre for Human Brain Health, School of Psychology, University of Birmingham, Birmingham, B15 2TT UK; 2 Child Neuropsychology Section, Department of Child and Adolescent Psychiatry, Psychosomatics and Psychotherapy, RWTH Aachen University, Aachen, Germany; 3 Department of Child and Adolescent Psychiatry, Faculty of Medicine, TU, Dresden, Germany; 4 School of Computer Science, University of Birmingham, Birmingham, UK; 5 Institute for Mental Health, School of Psychology, University of Birmingham, Birmingham, UK; 6 Department of Child and Adolescent Psychiatry, Psychosomatics and Psychotherapy, University Hospital RWTH Aachen, Aachen, Germany; 7 Department of Child and Adolescent Psychiatry, Psychosomatics and Psychotherapy, Goethe University, University Hospital Frankfurt, Frankfurt am Main, Germany; 8 Faculty of Education, Hamburg University, Hamburg, Germany; 9 Department of Child and Adolescent Psychiatry, VU University Medical Center, Amsterdam, the Netherlands; 10 Department of Child and Adolescent Psychiatry, Institute of Psychiatry, Psychology and Neuroscience, Kings College London, London, UK; 11 Child Development Institute, Toronto, Canada; 12 Psychiatric Service, Basurto University Hospital, Bilbao, Spain; 13 Department of Child and Adolescent Psychiatry, University Psychiatric Hospitals, University of Basel, Basel, Switzerland; 14 University Hospital Mutua Terrassa, Barcelona, Spain; 15 JARA-Brain Institute II, Molecular Neuroscience and Neuroimaging, RWTH Aachen and Research Centre Juelich, Juelich, Germany; 16 Department of Psychology, University of Bath, Bath, UK

**Keywords:** Emotion recognition, Conduct disorder, Conduct problems, Callous-unemotional traits, Machine learning

## Abstract

Conduct disorder (CD) with high levels of callous-unemotional traits (CD/HCU) has been theoretically linked to specific difficulties with fear and sadness recognition, in contrast to CD with low levels of callous-unemotional traits (CD/LCU). However, experimental evidence for this distinction is mixed, and it is unclear whether these difficulties are a reliable marker of CD/HCU compared to CD/LCU. In a large sample (*N* = 1263, 9–18 years), we combined univariate analyses and machine learning classifiers to investigate whether CD/HCU is associated with disproportionate difficulties with fear and sadness recognition over other emotions, and whether such difficulties are a reliable individual-level marker of CD/HCU. We observed similar emotion recognition abilities in CD/HCU and CD/LCU. The CD/HCU group underperformed relative to typically developing (TD) youths, but difficulties were not specific to fear or sadness. Classifiers did not distinguish between youths with CD/HCU versus CD/LCU (52% accuracy), although youths with CD/HCU and CD/LCU were reliably distinguished from TD youths (64% and 60%, respectively). In the subset of classifiers that performed well for youths with CD/HCU, fear and sadness were the most relevant emotions for distinguishing them from youths with CD/LCU and TD youths, respectively. We conclude that non-specific emotion recognition difficulties are common in CD/HCU, but are not reliable individual-level markers of CD/HCU versus CD/LCU. These findings highlight that a reduced ability to recognise facial expressions of distress should not be assumed to be a core feature of CD/HCU.

**Supplementary Information:**

The online version contains supplementary material available at 10.1007/s00787-021-01893-5.

## Introduction

Conduct disorder (CD) is a childhood psychiatric disorder consisting of severe and persistent aggression and violations of age-appropriate social norms. A subset of youths with CD also present with high levels of callous-unemotional (CU) traits (i.e., low levels of empathy and remorse), which are the core affective features of psychopathy [1]. This presentation is now recognised as a distinct subtype of CD [2], referred to here as CD/HCU. Youths with CD/HCU typically exhibit more severe and instrumental aggression than youths with CD and lower levels of CU traits (CD/LCU) [3, 4]. According to Blair’s neurocognitive model, instrumental aggression in CD/HCU results from amygdala hypo-reactivity to facial expressions of distress, specifically fear and sadness ([5]. For typically developing (TD) youths, the distress of others is aversive and inhibits aggression, but in CD/HCU, no such deterrence occurs [5]. Blair’s theory predicts that because distress cues are not as salient for youths with CD/HCU, they will have disproportionate difficulties in recognising facial expressions of distress. By contrast, there is no theoretical reason to hypothesise specific difficulties with distress recognition in CD/LCU.

Although emotion recognition difficulties are common in CD [6], the evidence that these difficulties are as predicted by Blair’s model is mixed. Emotion recognition difficulties are not universal in CD [6, 7], and while some studies implicate CU traits in this heterogeneity (e.g. [8]), others do not [9]. In this study, we first used univariate analyses to investigate whether CD/HCU and CD/LCU were associated with group-level differences in emotion recognition abilities, especially fear and sadness, relative to each other and to TD youths. We then used a multivariate machine learning classifier to examine whether, and to what extent, these differences provided reliable markers for distinguishing between CD/HCU, CD/LCU, and TD at the level of individual youths.

### Evidence for emotion recognition difficulties in CD and its CU trait subtypes

Mixed CD samples (where youths are not subtyped by CU traits) usually exhibit general difficulties in recognising emotions, which are not specific to fear and sadness, nor negative emotions more generally (e.g. [9] but [10, 11]). Using a very large sample overlapping with the one used in the present study, Kohls et al. [6] reported non-specific emotion recognition difficulties in CD. However, the effect size was small, highlighting that many youths with CD exhibit normative emotion recognition abilities (see also [7]). These studies suggest that general difficulties should be expected in both CD/HCU and CD/LCU, although these might be inconsistent across individuals. When CD/HCU and CD/LCU are considered separately, several studies report particular difficulties with negative emotions in CD/HCU (or “affective disturbance and impulsive and conduct problems”—[12])). CD/HCU has been associated with fear and sadness recognition difficulties over and above those seen in CD/LCU [8, 12–14], although one of these studies also reported difficulties with surprise [13]. Hartmann and Schwenck [15] reported that youths with CD/HCU exhibited similar accuracy to those with CD/LCU when identifying anger, sadness, and fear, but their reaction times were slower, suggesting a processing speed deficit (or perhaps an attentional deficit; see, e.g. [16]). Others have reported negative associations between CU or psychopathic traits and sadness, fear, and disgust recognition, as well as between antisocial behaviour and fear, anger, sadness, and disgust recognition [17, 18]. However, emotion recognition difficulties in CD/HCU are not consistently limited to negative emotions [19], nor consistently worse than in CD/LCU [7, 11, 20]. Kohls et al. [6] also found no evidence for a correlation between CU traits and emotion recognition abilities. Indeed, a positive association between CU traits and fear recognition was reported in a mixed CD-and-TD sample [21]. Thus, although there are indications that CD/HCU is linked to disproportionate difficulties in recognising negative emotions, especially fear and sadness, it is not yet clear how consistent this association is, especially across individuals.

### A machine learning approach

The principal advantages of a machine learning classifier in this context are its multivariate nature, combined with its ability to make predictions about individuals. By projecting data into multi-dimensional feature space (with each predictor variable, or feature, constituting a different dimension), a classifier is able to construct a decision boundary that optimally separates individuals of different classes within this space. In doing so, it naturally takes into account all features and their interactions simultaneously. This decision boundary is then used to predict the class membership (e.g. CD subtype) of previously unseen individuals, based on their location in the feature space. Furthermore, in-built feature selection or ranking procedures can provide additional information about the relative importance of each feature for classification, within the multivariate context. A machine learning approach therefore complements a standard univariate analysis, which is hypothesis-driven and uses an explicit statistical model to address a specific hypothesis. In this case, we use a univariate approach to determine whether or not there are group-level differences in emotion recognition abilities in CD/HCU and CD/LCU, especially for fear and sadness, and a machine learning classifier to quantify how reliable any such differences are as markers of CD subtype in individual youths.

The classifier used here, Angle-based Generalised Matrix Learning Vector Quantisation (Angle-GMLVQ; [22]), has the advantage of an in-built feature relevance procedure, which can be used to rank features for their relevance to the classification decision. In addition, Angle-GMLVQ is sensitive to the relative differences (angles) between features rather than their absolute magnitude. This makes it especially sensitive to different patterns of performance across emotions (e.g. specific difficulties with fear and sadness) rather than general emotion recognition difficulties. For an example of this approach, please see [23], where we used Angle-GMLVQ to classify CD/HCU, CD/LCU, and TD based on differences in parenting behaviours.

### Hypotheses

We used a large, well-characterised sample of youths with CD (FemNAT-CD) [24] to compare facial emotion recognition abilities in youths with CD/HCU, youths with CD/LCU, and TD youths. First, we hypothesised that general emotion recognition performance (i.e. across all emotions) would be poorest in youths with CD/HCU, followed by youths with CD/LCU and then TD youths. Second, we tested the hypothesis that youths with CD/HCU have specific difficulties with fear and sadness recognition, over and above any general difficulties. We also included sex as a factor in these analyses, to check whether any group differences were consistent across the sexes. These two hypotheses were investigated using univariate analyses (ANCOVA).

Next, we used Angle-GMLVQ to quantify the reliability of emotion recognition differences as markers of CD subtype. Although machine learning classification is not fundamentally a hypothesis-driven approach, we made several predictions about classifier performance based on our earlier hypotheses. First, based on group differences in performance, we predicted that classifier performance would exceed chance level (50% accuracy) when distinguishing between any two classes (e.g. CD/HCU from CD/LCU, CD/HCU from TD, etc.), and would be highest when distinguishing CD/HCU from TD. In line with our second hypothesis regarding CD/HCU and fear and sadness, we predicted that recognition of fear and sadness might be particularly relevant as markers of CD/HCU. Related to this prediction, we investigated whether a CD/HCU-against-TD and a CD/LCU-against-TD classifier would outperform a CD-against-TD classifier (where the CD group consisted of all youths with CD). If CD subtypes are indeed characterised by different patterns of difficulties, then distinguishing them from TD should be easier when the distinction between subtypes (i.e. CD/HCU vs. CD/LCU) is made.

## Methods

### Participants

Participants were selected from the FemNAT-CD dataset. Ethical approval details are provided in supplementary materials. Exclusion criteria were an IQ below 70, a history of manic or psychotic episodes, autism, or neurological or genetic disorders. In addition to these criteria, TD youths were also required to have no history of externalising disorders and no current psychiatric disorders.

Data on emotion recognition ability and CU traits were available for 1462 youths. Consistent with our recent work on this dataset [23], youths with CD were included in the CD/HCU group if their total score on the Inventory of Callous-Unemotional traits (ICU; [25]) was in the first tertile for youths with CD (a score of 39 or above), and in the CD/LCU group if their score was in the third tertile (29 or below). One hundred and ninety-nine participants with CD and second-tertile CU scores were excluded because they did not meet the criteria for either the CD/HCU or CD/LCU group. A small number of TD participants also had CU scores in the first tertile (*n* = 13) or second tertile (*n* = 44), but were included in the sample because they were typically developing according to the FemNAT-CD criteria, and excluding these participants did not change the pattern of results (analyses available from the authors on request). The final sample therefore consisted of 1263 participants: 248 with CD/HCU (153 females), 230 with CD/LCU (130 females), and 785 TD youths (523 females).^1^ For one of the Angle-GMLVQ classification analyses, the CD/HCU and CD/LCU groups were combined into a single CD group (*n* = 478), which we refer to as the ‘CD group’. Demographic differences between excluded and included participants are provided in supplementary materials.

### Questionnaire and interview measures

CD and other relevant disorders were assessed by trained researchers, via separate clinical interviews with participants and their parents or carers, using the Schedule for Affective Disorders and Schizophrenia in School-Age Children: Present and Lifetime Version (K-SADS-PL) [26]. Number of CD symptoms reflects the most serious lifetime episode. Where discrepancies arose between parent and youth ratings, interviewers discussed all available evidence to come to a best estimate. Other disorders assessed with the K-SADS-PL were attention-deficit/hyperactivity disorder, anxiety, depression, and substance use disorder. These disorders were controlled for in an additional set of analyses (see supplementary materials). To provide a more comprehensive clinical picture, we also report K-SADS-PL oppositional defiant disorder symptoms, although these did not form part of our analyses. CU traits were assessed with the ICU ([25]. This is a 24-item parent-report measure consisting of three subscales (callous, uncaring, and unemotional) plus a total score, which we used in the current study. Reliability for the ICU in the current sample was good (Cronbach’s α for total score = 0.93). Verbal, performance, and total IQ were estimated with the Wechsler Intelligence Scales [27, 28]. For completeness, we also report pubertal development stage as assessed with the Pubertal Development Scale (PDS) [29]. Socioeconomic status (SES) was assessed with a measure based on parental income, education level, and occupation, normalised for the country in which the participant lived (see [23]). Imputation of missing data is described in supplementary materials.

### Emotion hexagon task

**Fig. 1:**
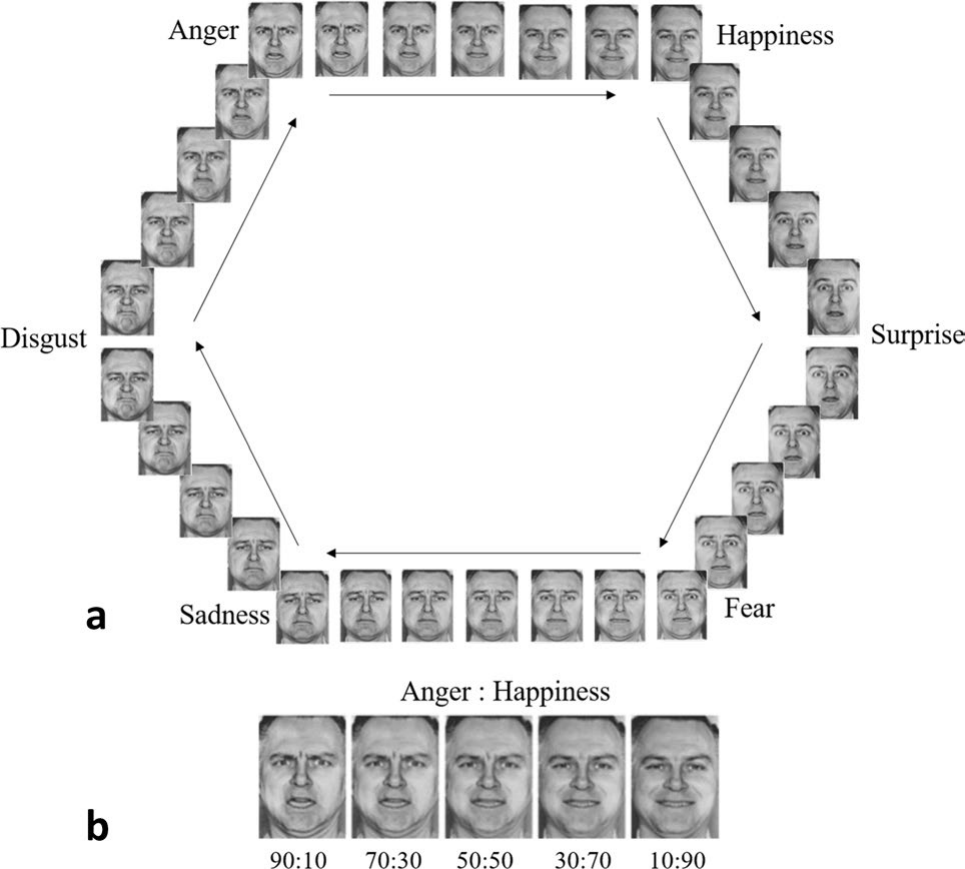
Stimuli used in the Emotion Hexagon Task. **a** The complete set of blended expressions arranged in a hexagon. The six basic emotions lie on the vertices adjacent to their most easily confused emotion (except anger–happiness, which adjoin to complete the hexagon). **b** An example of the blended expressions for the anger-to-happiness continuum, with ratios labelled

Emotion recognition ability was assessed with the Emotion Hexagon task [30]. Full details are given in supplementary materials. Briefly, participants were presented with blended expressions of two emotions, in the ratios of 90:10, 70:30, and 50:50 (see Fig. 1). Expressions were presented individually, and participants selected with a mouse click one of six labels (see Fig. 1a) that best described the expression. The ‘dominant’ emotion was considered the correct response. For example, ‘anger’ was the correct response to an expression that was a 90:10 or 70:30 blend of anger:happiness, while ‘happiness’ was the correct response to an expression that was a 30:70 or 10:90 blend of anger:happiness. 50:50 blends were not scored, as there was no ‘correct’ answer for these expressions. For each participant, we calculated the percentage recognition accuracy for each emotion at its highest intensity (i.e. its 90:10 ratio, or 90%, presentations) and its lowest (70:30, or 70%) intensity. We refer to these as the ‘high intensity’ and ‘low intensity’ of each emotion. We considered anger, disgust, fear, and sadness to be negative emotions [31], but only fear and sadness to be ‘distress’ emotions, in line with their theoretical role in CD/HCU [5].

### Univariate analysis

Interactions between group, emotion, intensity (high/low), and sex were investigated using a repeated measures ANCOVA (using IBM SPSS Statistics 25), with group and sex as between-subject factors and emotion and intensity as within-subject factors. Site of data collection was included as a factor of no interest, and grand-mean-centred age, total IQ, and SES were entered as covariates. Analysis of sex differences was exploratory, motivated by previous mixed evidence as to whether females with CD exhibit the same difficulties as males [6, 10].

### Classification analysis

The classification analyses used features derived from the 12 measures of Emotion Hexagon task performance (i.e. accuracy for six emotions at two intensity levels) to predict group status (i.e. classes were CD/HCU, CD/LCU, TD etc.). Since covariates cannot be controlled for in classification analyses, we regressed out variance associated with age, IQ, site of data collection, sex, and SES from each of the 12 measures of task performance. The final features were, thus, the standardised residual mean accuracies for each emotion at its high and low intensity levels. To assess the performance for each pair of groups of interest separately, four Angle-GMLVQ models were created with two classes each:

1.CD against TD (‘CD–TD’ model)
2.CD/HCU against TD (‘HCU–TD’ model)
3.CD/LCU against TD (‘LCU–TD’ model)
4.CD/HCU against CD/LCU (‘HCU–LCU’ model)

For each model, 100 separate classifiers were trained and tested using data re-sampling (see supplementary materials for a full description of the training and testing procedure). After training and testing each of the four models 100 times, mean performance measures were calculated for each model. Performance of the CD–TD, HCU–TD, and LCU–TD models were then compared using an ANOVA (the HCU–LCU model was not included in this ANOVA, because comparisons with the HCU–LCU model were not relevant to hypotheses). Model performance was assessed using macro-averaged accuracy (adapted from [32, 33]). This is the mean of the accuracies for each class, and is not skewed by superior performance for the larger class when class sizes are imbalanced. Positive predictive value (PPV), negative predictive value (NPV), true-positive rate (TPR), and true-negative rate (TNR) are also reported. These represent the proportion of positive classifications (e.g. CD/HCU) that are true positives (PPV), the proportion of negative classifications (e.g. TD) that are true negatives (NPV), the proportion of positives (i.e. cases) that are correctly classified as positives (TPR), and the proportion of negatives that are correctly classified as negatives (TNR).

### Feature relevance

The Angle-GMLVQ classifier generates a feature relevance score for each feature, for each trained classifier. The relevance score of a feature is a non-negative number that quantifies the importance of that feature for the given classification task. The relevance scores are normalised to sum to 1 across all features. In this way, the relevance scores learned in the 100 classifiers are directly comparable. The procedure described above thus generates 100 feature relevance scores for each feature, for each model. However, some of these classifiers will fail to distinguish between the groups, and the feature relevance scores from these models are, thus, not informative. We therefore discarded relevance scores from classifiers that did not achieve at least 50% macro-averaged accuracy. For each model, mean relevance scores across the retained classifier models were then calculated for each feature, and compared with a one-way ANOVA. We note that such feature score learning is effectively performing feature selection [34].

### Supplementary analyses

We conducted additional analyses to examine the correlations between CD symptoms, CU traits, and emotion recognition ability within each group. We also repeated the ANCOVAs and machine learning analyses after controlling for comorbid diagnoses (attention-deficit/hyperactivity disorder, depression, anxiety, and substance use disorder; note that youths with autism were excluded from this study). Finally, we investigated group differences in the number of remote prototype errors, that is, mislabelling an emotion as a non-adjacent emotion rather than an emotion with which the target was blended. Results of these analyses are reported in supplementary materials.

Finally, we also present results for two additional sets of classification analyses. First, we repeated the classification analyses described above with a support vector machine (SVM), to check that performances were comparable with this more established classifier. Second, we repeated the Angle-GMLVQ analyses using 23 instead of 12 features. These 23 features captured the percentage of trials in which each emotion at each intensity level was mislabelled as each of the other emotions (e.g. low-intensity anger mislabelled as happiness, low-intensity anger mislabelled as sadness, etc.).

## Results

### Demographic and clinical characteristics

Group differences in demographic and clinical characteristics are displayed in Table 1. All three groups differed in CD symptoms and CU trait severity, with the CD/HCU group exhibiting the most severe presentation, as expected. There were also group differences in the number of participants per group recruited from each site (*χ*^2^ = 48.00, *p* < 0.001, Φ_c_ = 0.14; see supplementary materials).

**Table 1:** Demographic and clinical characteristics (mean (95% confidence intervals of the mean) unless stated otherwise)

Measures	CD/HCU (*n* = 248)	CD/LCU (*n* = 230)	TD (*n* = 785)	Test statistic (p), effect size
Age (years)	14.14^a^ (13.86, 14.41)	14.30^a^ (13.98, 14.62)	13.95^a^ (13.78, 14.13)	*F* = 1.94 (0.14), η^2^_p_ = 0.003
Females (%)	61.69^a,b^	56.52^a^	66.62^b^	*χ*^2^ = 8.46 (0.02), Φ_c_ = 0.08
SES	− 0.38^a^ (− 0.51, − 0.26)	− 0.29^a^ (− 0.41, − 0.16)	0.33^b^ (0.26, 0.39)	*F* = 70.29 (< 0.001), η^2^_p_ = 0.11
PDS pubertal stage	3.60^a^ (3.47, 3.72)	3.55^a^ (3.41, 3.7)	3.52^a^ (3.44, 3.59)	*F* = 0.53 (0.59), η^2^_p_ = 0.001
Performance IQ	97.21^a^ (95.31, 99.11)	96.74^a^ (94.77, 98.7)	103.37^b^ (102.29, 104.45)	*F* = 25.56 (< 0.001), η^2^_p_ = .04
Verbal IQ	92.83^a^ (91.1, 94.57)	92.89^a^ (90.9, 94.87)	103.52^b^ (102.37, 104.66)	*F* = 67.27 (< 0.001), η^2^_p_ = 0.10
Total IQ	95.26^a^ (93.76, 96.76)	94.95^a^ (93.26, 96.63)	103.73^b^ (102.79, 104.67)	*F* = 63.93 (< 0.001), η^2^_p_ = 0.09
ICU callous	18.15^a^ (17.54, 18.75)	6.40^b^ (5.96, 6.84)	4.28^c^ (4.05, 4.5)	*F* = 1417.01 (< 0.001), η^2^_p_ = .70
ICU uncaring	18.33^a^ (17.98, 18.69)	10.20^b^ (9.69, 10.71)	7.74^c^ (7.45, 8.03)	*F* = 703.87 (< 0.001), η^2^_p_ = 0.53
ICU unemotional	9.73^a^ (9.36, 10.09)	5.11^a^ (4.76, 5.46)	4.83^b^ (4.64, 5.03)	*F* = 297.88 (< 0.001), η^2^_p_ = 0.32
ICU total	46.21^a^ (45.4, 47.01)	21.71^b^ (20.96, 22.46)	16.84^c^ (16.29, 17.39)	*F* = 1553.34 (< 0.001), η^2^_p_ = 0.71
CD symptoms*	5.78^a^ (5.48, 6.08)	5.03^b^ (4.69, 5.38)	0.09^c^ (0.06, 0.11)	*F* = 1753.42 (< 0.001), η^2^_p_ = 0.74
ODD symptoms*	6.41^a^ (6.17, 6.64)	5.31^b^ (5.02, 5.6)	0.08^c^ (0.06, 0.11)	*F* = 2926.52 (< 0.001), η^2^_p_ = 0.82
ADHD symptoms*	7.83^a ^(6.97, 8.69)	6.03^b^ (5.21, 6.85)	0.11^c^ (0.07, 0.15)	*F* = 428.60 (< 0.001), η^2^_p_ = 0.41
GAD diagnosis (%)*	17.74^a^	15.65^a^	1.91^b^	*χ*^2^ = 94.63 (< 0.001), Φ_c_ = 0.27
MDD diagnosis (%)*	25.00^a^	23.91^b^	1.66^b^	*χ*^2^ = 167.72 (< 0.001), Φ_c_ = 0.36
SUD diagnosis (%)*	21.77^a^	17.39^a^	0.13^b^	*χ*^2^ = 166.33 (< 0.001), Φ_c_ = 0.36

*CD/HCU* conduct disorder with high levels of callous-unemotional traits. *CD/LCU* conduct disorder with low levels of callous-unemotional traits. *TD* typically developing. *PDS* Pubertal Development Scale (self-report). ICU, Inventory of Callous-Unemotional Traits. *SES* socioeconomic status. *CD* conduct disorder. *ODD* oppositional defiant disorder. *ADHD* attention-deficit/hyperactivity disorder. *GAD* generalised anxiety disorder. *MDD* major depressive disorder. *SUD* substance use disorder. *η2p*, partial eta squared. Φc, Cramer’s phi. Groups with different superscript indices differ significantly in post hoc comparisons (*p* < 0.05, Bonferroni corrected)

*Assessed using K-SADS, Schedule for Affective Disorders and Schizophrenia for School-age Children: Present and Lifetime Version (lifetime maximum symptoms/diagnosis)

### Group differences in emotion recognition

**Fig. 2:**
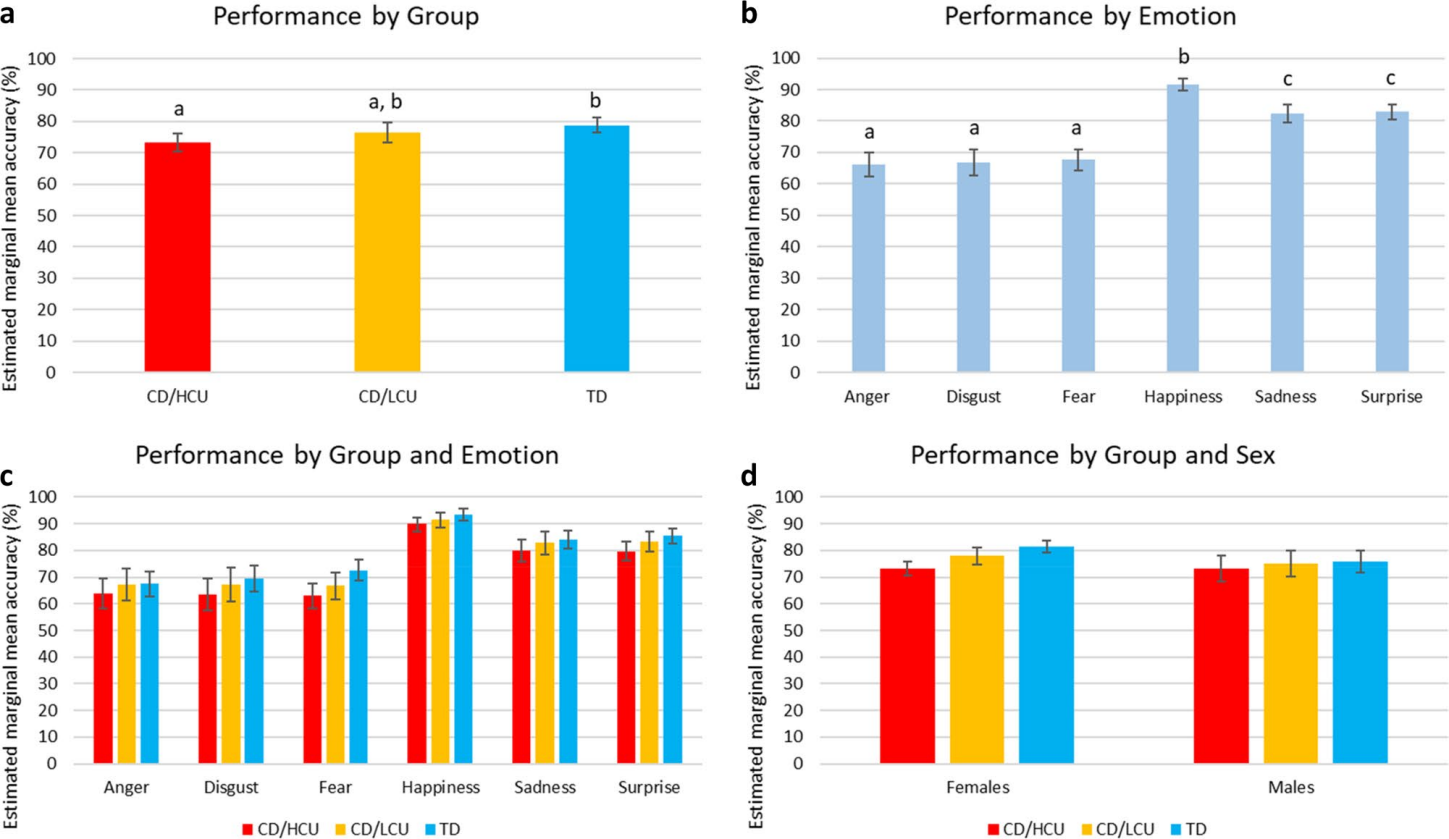
Emotion Hexagon recognition task performance by (**a**) emotion, (**b**) group, (**c**) group and sex, and (**d**) group and emotion. Different letters (**a**, **b**, **c**) above columns indicate significant differences in post hoc pairwise comparisons (*p* < 0.05, Bonferroni corrected). Pairwise comparisons were not conducted for panels c) and d) due to the absence of significant interactions. Error bars represent 95% confidence intervals of the mean

The ANCOVA revealed a significant main effect of group (F_(2, 1130)_ = 11.12, *p* < 0.001, η^2^_p_ = 0.02), as illustrated in Fig. 2a. The CD/HCU group performed significantly worse than the TD group, but the CD/LCU group did not differ from either group. Given this overlap, we repeated the ANCOVA with both CD subtypes combined into one group (i.e. CD versus TD) to check whether CD as a whole was associated with poorer emotion recognition. The second ANCOVA confirmed that youths with CD exhibited poorer performance than TD youths (F_(1, 1141)_ = 21.62, *p* < 0.001, η^2^_p_ = 0.02).

There was also a significant main effect of emotion (F_(3.71, 4190.00)_ = 55.5, *p* < 0.001, η^2^_p_ = 0.05), as illustrated in Fig. 2b. Across the full sample, recognition accuracy was highest for happiness, followed by sadness and surprise, and then anger, disgust, and fear. However, the interaction between group and emotion was not significant (F_(7.42, 4190.99)_ = 0.62, *p* = 0.75, η^2^_p_ = 0.001), suggesting that the underperformance of the CD/HCU group was not disproportionate for fear and sadness (or indeed negative emotions more generally; Fig. 2c).

We did not formulate a priori hypotheses regarding emotion intensity, but noted that recognition accuracy was better for high-intensity than for low-intensity emotions (F_(1, 1130)_ = 80.51, *p* < 0.001, η^2^_p_ = 0.07). Interestingly, the performance gap between the CD/HCU, CD/LCU, and TD groups widened slightly for high-intensity emotions (group by intensity interaction: F_(2, 1130)_ = 3.66, *p* = 0.03, η^2^_p_ = 0.01), suggesting that the TD group, and to a lesser extent also the CD/LCU group, benefited more from the increased expression intensity than the CD/HCU group. However, the three-way interaction between group, emotion, and intensity was not significant (F_(9.24, 5218.77)_ = 1.33, *p* = 0.21, η^2^_p_ = 0.002), confirming that this pattern did not reflect specific difficulties with negative emotions at either intensity in the CD/HCU group.

There were small but significant correlations between age and sex (Pearson correlation: *r* =  − 0.09, *p* = 0.003) and between sex and total IQ (*r* = 0.07, *p* = 0.02), with boys tending to be younger than girls and to have higher IQs. Consequently, sex effects must be interpreted with extreme caution. However, investigation of sex differences revealed that female youths significantly outperformed male youths, as evidenced by a main effect of sex (F_(1, 1130)_ = 5.88, *p* = 0.02, η^2^_p_ = 0.005). Although differences between the CD/HCU, CD/LCU, and TD groups were slightly larger for females than males (Fig. 2d), this group by sex interaction was not significant (F_(2, 1130)_ = 0.44, *p* = 0.65, η^2^_p_ = 0.001), confirming that the pattern of group differences was similar for both sexes. In summary, in these univariate analyses, group differences in emotion recognition abilities were not driven by difficulties with specific emotions such as fear and sadness.

### Classifier performance

We first compared performance for the CD–TD, HCU–TD, and LCU–TD models (Table 2). Only the HCU–TD model achieved higher accuracy than the CD–TD model, with the LCU–TD model achieving the lowest accuracy of these three models. Nonetheless, all performed significantly above chance (i.e. 50%; binomial tests: *p* < 0.001, *p* < 0.001, and *p* = 0.002, respectively). Notably, TD youths were consistently more likely to be classified correctly (TNRs) than youths with CD/HCU or CD/LCU (TPRs), suggesting ‘normative’ performance in approximately 50% of youths with CD regardless of CU traits. Finally, the performance of the HCU–LCU model was at chance level (52%; binomial test, *p* = 0.42), indicating that emotion recognition difficulties were not a reliable marker of subtype within CD. The pattern of results was similar for the additional sets of classification analyses, using an enlarged set of 23 features for the Angle-GMLVQ classifier and repeating the main analyses with SVM classifiers (see supplementary materials).

**Table 2:** Angle-GMLVQ model performance (mean (95% confidence intervals of the mean))

	CD–TD	HCU–TD	LCU–TD	F (p), η^2^_p_	HCU–LCU
Accuracy (macro-averaged)	0.62^a^ (0.62, 0.63)	0.64^b^ (0.64, 0.65)	0.60^c^ (0.60, 0.61)	33.69 (< 0.001), .19	0.52 (0.51, 0.53)
PPV	0.54^a^ (0.53, 0.55)	0.40^b^ (0.39, 0.41)	0.33^c^ (0.33, 0.34)	718.60 (< 0.001), .83	0.53 (0.52, 0.54)
NPV	0.71^a^ (0.71, 0.72)	0.84^b^ (0.83, 0.84)	0.83^c^ (0.83, 0.83)	1123.18 (< 0.001), .88	0.50 (0.49, 0.51)
TPR	0.51^a^ (0.50, 0.52)	0.54^b^ (0.52, 0.55)	0.51^a^ (0.49, 0.52)	6.64 (0.002), 0.04	0.45 (0.43, 0.46)
TNR	0.74^a^ (0.73, 0.74)	0.75^a^ (0.74, 0.75)	0.70^b^ (0.69, 0.71)	34.14 (< 0.001), 0.19	0.59 (0.57, 0.60)

*CD–TD *conduct disorder–typically developing model. *HCU–TD* high callous-unemotional–typically developing model. *LCU–TD* low callous-unemotional–typically developing model. *PPV* positive predictive value. *NPV* negative predictive value. *TPR* true-positive rate. *TNR* true-negative rate. η2p partial eta squared. Groups with different superscript indices differ significantly in post hoc comparisons (*p*<0.05, Bonferroni corrected). Note that the HCU−LCU model (column 6) was not included in statistical tests as comparisons between this and other models were not relevant to hypotheses

### Feature relevance

**Fig. 3:**
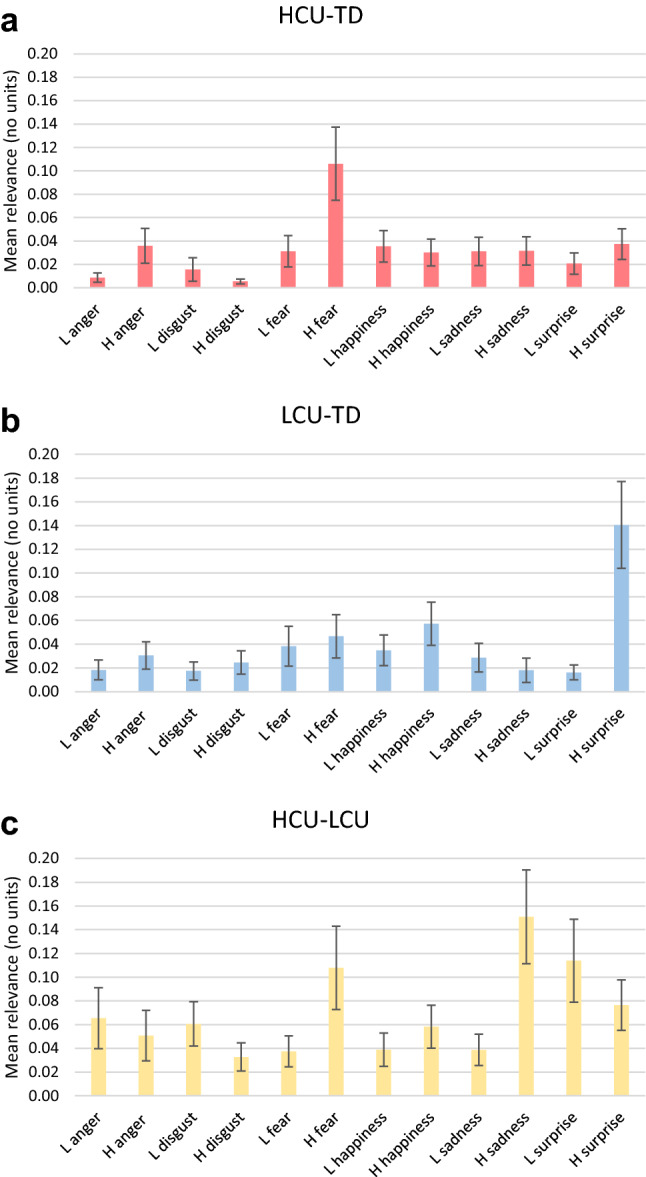
Mean feature relevance scores for (**a**) the HCU–TD model, (**b**) LCU–TD model, and (**c**) HCU–LCU model. Error bars represent 95% confidence intervals of the mean. *L* = low intensity, *H* = high intensity

Mean feature relevance scores for the HCU–TD, LCU–TD, and HCU–LCU models are displayed in Fig. 3. Feature relevance scores were not normally distributed and were thus compared with Kruskal–Wallis (non-parametric) ANOVAs. For the HCU–TD model, the most relevant feature was high-intensity fear, which differed significantly from all other emotions (H_(11)_ = 139.2, *p* < 0.001: post hoc Bonferroni-corrected comparison of mean ranks, all *p* < 0.05). For the LCU–TD model, the most relevant feature was high-intensity surprise, which was significantly more relevant than all other features except high-intensity happiness (H_(11)_ = 101.49, *p* < 0.001: post hoc Bonferroni-corrected comparison of mean ranks, all *p* < 0.05 except comparison with high-intensity happiness). For the HCU–LCU model, the most relevant feature was high-intensity sadness, which was significantly more relevant than anger (both intensities), high-intensity disgust, and low-intensity fear, happiness, and sadness (H_(11)_ = 51.93, *p* < 0.001: post hoc Bonferroni-corrected comparison of mean ranks, *p* < 0.05).

## Discussion

The aim of this study was to investigate whether CD/HCU and CD/LCU are associated with differences in emotion recognition abilities, especially for fear and sadness, and whether any such difficulties are sufficiently reliable to act as markers for CD subtype in individual youths. First, we hypothesised that overall emotion recognition ability would be lowest in youths with CD/HCU, with youths with CD/LCU performing at an intermediate level between youths with CD/HCU and TD youths. Evidence supporting this hypothesis was somewhat weak. The direction of group differences was as predicted, but only the CD/HCU and TD groups differed significantly in univariate analyses. Despite this, and although classifier performance was not strong, the Angle-GMLVQ classifier was able to discriminate both youths with CD/HCU and youths with CD/LCU from TD youths at above-chance accuracy levels. Second, we hypothesised that CD/HCU would be associated with particular difficulties with fear and sadness. Evidence for this hypothesis was very weak. There was no group by emotion interaction in the univariate analysis, and the CD–TD classifier performed less well than the HCU–TD classifier only, suggesting that differences between CD/HCU and CD/LCU were mostly quantitative rather than qualitative. However, the most relevant features in the HCU–TD and HCU–LCU models were fear and sadness, respectively; whereas surprise was most relevant for the LCU–TD model. Difficulties with fearful and sad facial expressions were, thus, the strongest markers of CD/HCU, but not CD/LCU, in individual youths.

### Prevalence of emotion recognition difficulties in CD and implications for its theoretical understanding

These findings are in line with previous meta-analytic work [19], and suggest that facial emotion recognition difficulties are common in CD/HCU and are generally not restricted to fear and sadness. In contrast to previous findings from an all-female sample [10], but in line with work by Kohls et al. [6] and Fairchild et al. [14], difficulties were seen in female as well as male youths. However, emotion recognition difficulties in CD were by no means universal, especially for the CD/LCU group, who did not differ significantly from the TD group in univariate analyses. Indeed, the effect size for the main effect of group in the univariate analyses was small, and in multivariate analyses, approximately 50% of youths with CD were misclassified. By contrast, TD youths were misclassified far less frequently. This pattern of misclassification implies an ‘imbalanced’ overlap between groups, with youths in the CD groups more likely to resemble TD youths than vice versa. These findings suggest that while poor emotion recognition ability is considerably more common in CD than in TD youths, many youths with CD exhibit ‘normal' emotion recognition abilities, in line with recent research in this area [6, 7, 10].

Group-level differences between CD/HCU and CD/LCU did not emerge in the present study, in line with a minority of previous studies [7, 11]. These divergent findings might be partially explained by differences in study design, criteria for CD/HCU, and choice of control group in previous research (CD/LCU or TD youths). However, studies that do report poorer emotion recognition in CD/HCU often report particular difficulties with negative emotions, especially fear and sadness [12–14]. This is notable because in our Angle-GMLVQ analyses, fear and sadness recognition difficulties were the most relevant markers of CD/HCU, despite the lack of significant group differences. Feature relevance scores were based on classifiers that achieved at least 60% classification accuracy, which implies that when the classifiers *were* able to discriminate between CD/HCU and the other groups, they did so principally on the basis of fear and sadness recognition. This finding may help to clarify the previous literature, where group differences are not always present, but when they are, they are strongest for fear and sadness.

Importantly, these results also highlight a discrepancy between theoretical descriptions of CD/HCU, namely that these are youths who have an impaired ability to recognise and respond appropriately to distress in others [5], and common clinical or research definitions of CD/HCU, which are based directly on high scores on CU trait measures. The latter are agnostic with regard to aetiology and underlying neurocognitive characteristics. The lack of precise clinical criteria and measures for CD/HCU is obviously a major hindrance to the literature here, since it is possible that the discrepancy between clinical presentation (CD/HCU) and the assumed underlying psychopathology (emotion recognition deficits) simply reflects an inappropriate threshold for CD/HCU. This might be the case if CU traits and emotion recognition have a non-linear relationship, with emotion recognition deficits manifesting only at the extreme upper end of the CU trait spectrum (see, e.g. [35]). Alternatively, if the relationship is linear, the precise cut-off is less crucial. However, we note that an approach based more closely on the Diagnostic and Statistical Manual of Mental Disorders Limited Prosocial Emotions criteria, and using a sample that overlaps with the present one, also failed to identify any association between CU traits and emotion recognition performance [7]. These considerations around clinical threshold notwithstanding, the results of the current study raise a broader question as to whether CU traits constitute the psychopathology itself (thus, warranting a behavioural diagnosis), or are an indirect measure of an underlying psychopathology such as emotion recognition deficits. Our findings do not support any particular position on this point, but they do highlight the possibility that these two approaches will not identify the same subset of youths. Similar discrepancies were previously observed by Kohls et al. [6, 7] in the context of CD more generally, and they merit serious consideration both for future research and clinical approaches to CD.

### Emotional intensity and recognition accuracy

Finally, an interesting and unexpected finding in the present study was the pattern of feature relevance for the different emotion intensities in the classifier models. Somewhat counterintuitively, group differences in recognition accuracy were larger for the high-intensity emotions compared to the low-intensity emotions, and the feature relevance analyses confirmed that difficulties with high-intensity emotions were more relevant markers of CD subtypes than were low-intensity emotions. The reason for this is not clear, and further research with a wider range of emotional intensities [18, 36], as well as neutral faces, will be needed to confirm this finding. However, it suggests that TD youths benefit more from increased emotional intensity than do youths with CD, perhaps indicating that the difficulties seen in CD are not ‘threshold’ effects but rather more absolute difficulties with recognising emotions.

### Strengths and limitations

This study has several strengths, including a large, well-characterised, mixed-sex sample and the use of multivariate analyses. However, some limitations should be noted. First, the Emotion Hexagon task uses static expressions, and the identity of the face is the same across all expressions. The ecological validity of the task would be improved by the inclusion of a range of facial identities, as well as dynamic stimuli (although recent work indicates similar effects for dynamic stimuli [21]). Second, the Emotion Hexagon task does not include neutral expressions, which complicates the interpretation of errors. Third, the CD/HCU group in the current sample also had more severe CD than the CD/LCU group. Although this is clinically typical of CD/HCU, it is an important consideration, because some neuroimaging studies of amygdala reactivity to emotional faces report a stronger association with CD severity than with CU traits [37]. Fourth, TD participants had no current psychiatric disorders, and were, thus, not fully representative of the general population. This is notable due to the complex associations between internalising disorders and emotion recognition difficulties [36, 38]. Although we repeated our analyses after controlling for comorbidities and found no substantial changes, the nature of our TD group might still have led to an over- or underestimate of ‘normative’ emotion recognition abilities. Fifth, the sample spanned a large age range (9–18 years), and we were not able to investigate any potential developmental differences across this age range. Finally, we would have ideally validated our classifiers by further testing them on a completely independent dataset, but this was not possible due to a lack of available datasets.

## Summary and conclusions

In summary, these findings indicate that, compared to TD youths, youths with CD/HCU frequently have difficulty recognising facial emotional expressions. However, differences between CD/HCU and CD/LCU were smaller and not significant. Indeed, there was substantial overlap between all three groups, emphasising again the heterogeneous nature of CD as a diagnostic category. Nonetheless, the most relevant emotions for distinguishing CD/HCU from TD and from CD/LCU in classification analyses were fear and sadness, indicating that when youths with CD/HCU do differ from other youths, they tend to differ most on recognition of fear and sadness. These findings highlight the existence of heterogeneous abilities within both CD/HCU and CD/LCU, and the need to establish what (if any) level of difficulty is reliably associated with CD, and CD/HCU in particular. Longitudinal research designs will also be necessary to elucidate whether, or how, emotion recognition difficulties contribute to the development of antisocial behaviour and aggression in adolescence.

## Supplementary Information

Below is the link to the electronic supplementary material.

Supplementary file1 (DOCX 366 KB)

## Data Availability

Data are available from the authors on request.

## Footnotes

1 Of the 1263 participants in the current study, 1081 participants (372 with CD) were included in the studies by Kohls et al. [6], [7] and 182 (106 with CD) were not.

## Code availability

Code is available from the authors on request.
