# Supplementary material for: Machine learning classification of conduct disorder with high versus low levels of callous-unemotional traits based on facial emotion recognition abilities
Source: Eur Child Adolesc Psychiatry. 2021 Oct 18;32(4):589–600. doi: 10.1007/s00787-021-01893-5 (PMC10115711; doi:10.1007/s00787-021-01893-5)
Supplement: Supplementary file 1 — Supplementary file1 (DOCX 366 KB) [file 787_2021_1893_MOESM1_ESM.docx]

Supplementary Materials: Machine learning classification of conduct disorder with high versus low levels of callous-unemotional traits based on facial emotion recognition abilities (Pauli et al., 2021)

## Ethical Approval

The FemNAT-CD project received ethical approval from the relevant local ethics committees, as follows: Aachen: Ethik Kommission Medizinische Fakultät der Rheinisch Westfälischen Technischen Hochschule Aachen (EK027/14). Amsterdam: Medisch Etische Toetsingscommissie (2014.188). Athens: Election Committee of the First Department of Psychiatry, Eginition University Hospital (641/9.11.2015). Barcelona: Child and Adolescent Mental Health - University Hospital Mutua Terrassa (acta 12/13). Basel: Ethik Kommission Nordwest- und Zentralschweiz (EKNZ 336/13). Bilbao: Hospital del Basurto. Birmingham and Southampton: University Ethics Committee and National Health Service Research Ethics Committee (NRES Committee West Midlands, Edgbaston; REC reference 3/WM/0483). Dublin: SJH/AMNCH Research Ethics Committee (2014/04/Chairman (3)). Frankfurt: Ethik Kommission Medizinische Fakultät Goethe Universität Frankfurt am Main (445/13). Szeged (Hungary): Egészségügyi Tudományos Tanács Humán Reprodukciós Bizottság (CSR/039/00392-3/2014).

## Demographic differences between Included and Excluded Participants

Excluded and included participants did not differ significantly in age (t _(1463)_ = 1.94, *p* = .05), or proportion of females (χ^2^ _(1)_ = .02, *p* = .89), but excluded participants had lower total IQ scores (t _(1431)_ = -5.87, *p* < .001). For youths with CD, excluded and included participants did not differ significantly in number of CD symptoms (t _(676)_ = .21, *p* = .84) or level of CU traits (t _(551.26)_ = -.31, *p* = .76).

## Imputation of Missing Data

Missing data were imputed by statisticians at the Institute of Medical Biometry and Statistics (IMBI), a member of the FemNAT-CD consortium. Missing data for the PDS were imputed separately, before the decision was made to impute missing values for other measures. The procedure for the PDS imputation is thus described separately from the other measures. The following description is a standard text provided by IMBI, for use in all FemNAT-CD consortium publications.

Missing values of the PDS score were imputed based on the whole FemNAT-CD sample. It has been shown that missing data in a multi-item instrument is best handled by imputation at the item level (Eekhout, de Vet, Twisk, Brand, de Boer, & Heymans, 2014). Thus, missing values of the single items were imputed first, and the scores were calculated based on the imputed items. The imputation was done in SAS^®^ version 9.4 using the procedure PROC MI. Imputation by fully conditional specification (FCS) is used, which offers a flexible method to specify the multivariate imputation model for arbitrary missing patterns including both categorical and continuous variables (Liu & De, 2015). As the items are measured at an ordinal level, the logistic regression method is specified in the FCS statement. For imputation diagnostics, distribution of the observed and imputed items and scores were checked. The imputation of the PDS items was done separately in males and in females because of sex specific items: item 2 (females and males) and items 4, 5a of the form for females or items 4, 5 of the form for males were imputed respectively. The following variables were included in the imputation model: sex specific items of the PDS as mentioned above and the two remaining PDS items (items 1 and 3), age at PDS and age at informed consent, to impute age at PDS if missing, weight, case/control status, site, and migration status.

Imputation for the remaining measures was conducted separately, following the same procedure as above. The following variables were included in the imputation model: all items of the respective questionnaire, age, IQ, group (case/control), sex (male/female), site, comorbidities (post-traumatic stress disorder (PTSD), attention-deficit/hyperactivity disorder (ADHD), oppositional defiant disorder (ODD), depression, anxiety), and items of other questionnaires if correlated with at least one of the items with ≥.4. For imputation diagnostics, distribution of the observed and imputed items and scores were checked.

## Hexagon Task

Stimuli in the Hexagon task consist of ‘blended’ faces, each displaying combinations of two of the six basic emotions (anger, disgust, fear, happiness, sadness, and surprise) in intensity ratios of 90:10, 70:30, 50:50, 30:70 and 10:90. The blended expressions were presented on a computer monitor in random order for five seconds each and participants selected the label that best described the emotion presented from a list of the six emotions. There was no time limit for responding and no feedback was provided. Participants completed a practice block followed by five experimental blocks. Each of the 30 blended expressions was presented once per block. Correct responses were coded as those where the dominant emotion was selected (50:50 expressions were not scored). Percentage recognition accuracy was then calculated for each expression at its two ‘dominant’ intensity levels, i.e., high intensity disgust, low intensity disgust, high intensity happiness etc. This resulted in 12 emotion by intensity trial types (six emotions by two intensities).

## Angle-GMLVQ Model Training Procedure

The procedure for training and testing each of the individual classifiers was as follows: first, the data were split into a training set (80% of the data) and a testing set (20% of the data). This was a stratified split, i.e., preserving the original class proportions in both the training and testing sets. The class sizes in the training set were then equalised by randomly down-sampling the larger class to the size of the smaller class, to prevent bias in favour of the larger class during training. Next, both classes were randomly down-sampled to 90% of the size of the smaller class; this ensured that, in models where the class sizes were very similar at the outset, there was still variation in the data that went into each classifier. Finally, the classifier was trained and tested. This down-sampling procedure was repeated 100 times, yielding 100 different versions of the same classifier type.

## Correlations Between CD, CU Traits, and Emotion Recognition Accuracy

We calculated Pearson correlation coefficients between CD symptoms, CU traits, and mean emotion recognition accuracies (residualised) separately for the CD/HCU, CD/LCU, and TD groups. (Given that youths in the second CU tertile were removed from the CD group in this sample, correlations across the full CD group would largely reflect the selection criteria only.) There were no significant correlations between emotion recognition and CU traits in any group (*p* > .05). CD symptoms were negatively correlated with anger recognition in the CD/LCU group (*r* = -.14, *p* = .03) and with disgust (*r* = -.14, *p* = .03) and surprise (*r* = -.16, *p* = .01) in the CD/HCU group. We note, however, that the group design and associated selection procedure mean that correlations in this sample are not necessarily reflective of the complete FemNAT-CD sample, and must be interpreted with caution.

## Group Differences in Emotion Recognition after Accounting for Comorbidities

Inclusion of attention-deficit/hyperactivity disorder, depression, anxiety, and substance use disorder diagnoses as additional factors of no interest in the ANCOVA did not affect the principal findings. The main effect of group persisted (F _(2, 1126)_ = 13.23, *p* < .001, η^2^_p_ = .02), with the CD/HCU group underperforming relative to the TD group (*p* < .05, Bonferroni corrected). There was a main effect of emotion, similar to the main analyses (F _(3.71, 4173.68)_ = 29.14, *p* < .001, η^2^_p_ = .03), with accuracy highest for happiness, followed by sadness, then surprise, and then disgust, fear, and anger (not significant). Again, the group by emotion interaction was not significant (F _(7.41, 4173.68)_ = .40, *p* = .91, η^2^_p_ = .001).

Angle-GMLVQ classifier performance is shown in Table S1 and feature relevance scores are shown in Figure S1. Classification accuracy was lower after accounting for comorbidities, suggesting that some of the variation between CD and TD groups could be attributed to comorbid diagnoses in the CD group. There were also some changes to feature relevance scores, for example with high intensity anger becoming more relevant to the HCU-TD model than in our main analyses. However, the relevance scores remained largely in line with our theoretical interpretation of the main analyses. With these exceptions, results were largely unchanged from the main analyses.

**Table S1.** Angle-GMLVQ model performance after accounting for comorbidities (mean (95% confidence intervals of the mean))

|  | CD-TD | HCU-TD | LCU-TD | F (p), η^2^_p_ | HCU-LCU |
| --- | --- | --- | --- | --- | --- |
| Accuracy (macro-averaged) | 0.56 ^a^  (0.55, 0.56) | 0.57 ^b^  (0.57, 0.58) | 0.55 ^a^  (0.54, 0.55) | 15.40 (<.001), .09 | 0.51  (0.50, 0.52) |
| PPV | 0.45 ^a^  (0.44, 0.45) | 0.31 ^b^  (0.30, 0.32) | 0.27 ^c^  (0.26, 0.28) | 617.42 (<.001), .81 | 0.53  (0.52, 0.54) |
| NPV | 0.67 ^a^  (0.66, 0.67) | 0.81 ^b^  (0.80, 0.81) | 0.80 ^b^  (0.80, 0.80) | 1112.65 (<.001), .88 | 0.49  (0.48, 0.50) |
| TPR | 0.46 ^a^  (0.45, 0.47) | 0.49 ^b^  (0.48, 0.51) | 0.47 ^a^  (0.45, 0.48) | 5.85 (.003), .04 | 0.47  (0.45, 0.48) |
| TNR | 0.65 ^a^  (0.64, 0.66) | 0.66 ^a^  (0.65, 0.67) | 0.63 ^b^  (0.62, 0.64) | 14.12 (<.001), .09 | 0.55  (0.54, 0.57) |

^Notes: CD-TD, conduct disorder – typically developing model. HCU-TD, high callous-unemotional – typically developing model. LCU-TD, low callous-unemotional – typically developing model. PPV, positive predictive value. NPV, negative predictive value. TPR, true positive rate. TNR, true negative rate. η2p, partial eta squared. Groups with different superscript indices differ significantly in post-hoc comparisons (^*^p^* ^< .05, Bonferroni corrected). Note that the HCU-LCU model (column 6) was not included in statistical tests as comparisons between this and other models were not relevant to hypotheses^

**Figure S1.** Mean feature relevance scores for a) the HCU-TD model, b) LCU-TD model, and c) HCU-LCU model after regressing out variance associated with comorbidities

## Remote Prototype Errors

Remote prototype errors are errors in which emotions are mislabelled as non-adjacent emotions. These errors are ‘complete’ errors in the sense that they cannot be attributed to oversensitivity to the minority emotion in a blended expression. At the individual participant level, we calculated the percentage of responses to each emotion-by-intensity trial type where the response was a remote prototype error. For example, we calculated the percentage of remote prototype errors for low intensity anger by finding the percentage of low intensity anger trials that were labelled as fear, sadness, or surprise by the participant. Next, we averaged across the low and high intensity trials for each emotion, to give a mean percentage of remote prototype errors for each emotion. To avoid confounding the percentage of remote prototype errors with the percentage of total errors, we also calculated the percentage of ‘near-prototype’ errors using the same procedure. (For example, near prototype errors for anger trials are those that were labelled as happiness or disgust). Group differences in error type (remote versus near prototype) were then compared using an ANCOVA, with group as the between-subjects factor and emotion and error type as the between-subjects factor. For consistency with the main analyses, we entered sex and site of data collection as between-subjects factors of no interest, and mean-centred age, total IQ, and SES as covariates. Differences in error type by group and emotion are displayed in Figure S2.

Predictably, there was a main effect of error type, with near prototype errors being more common than remote prototype errors (F _(1.00, 1116.00)_ = 33.50, *p* < .001, η^2^_p_ = .013). There was also a significant emotion by error type interaction (F _(3.45, 3844.98)_ = 10.36, *p* < .001, η^2^_p_ = .01); there were significantly more near than remote prototype errors for disgust, fear, sadness, and surprise, no difference for anger, and significantly more remote than near prototype errors for happiness. Presumably, this reflects the fact that anger and happiness are the only two emotions that are not blended with their most easily confused neighbour.

Importantly, neither the group by error type interaction (F _(2.00, 1116.00)_ = 1.92, *p* = .15, η^2^_p_ = .003) nor the group by emotion by error type interaction (F _(6.89, 3844.98)_ = 1.55, *p* = .15, η^2^_p_ = .003) were significant. Thus, the CD/HCU group did not appear to have a particular tendency to make remote prototype errors for negative emotions, nor indeed to make relatively more remote than near prototype errors compared to other groups.


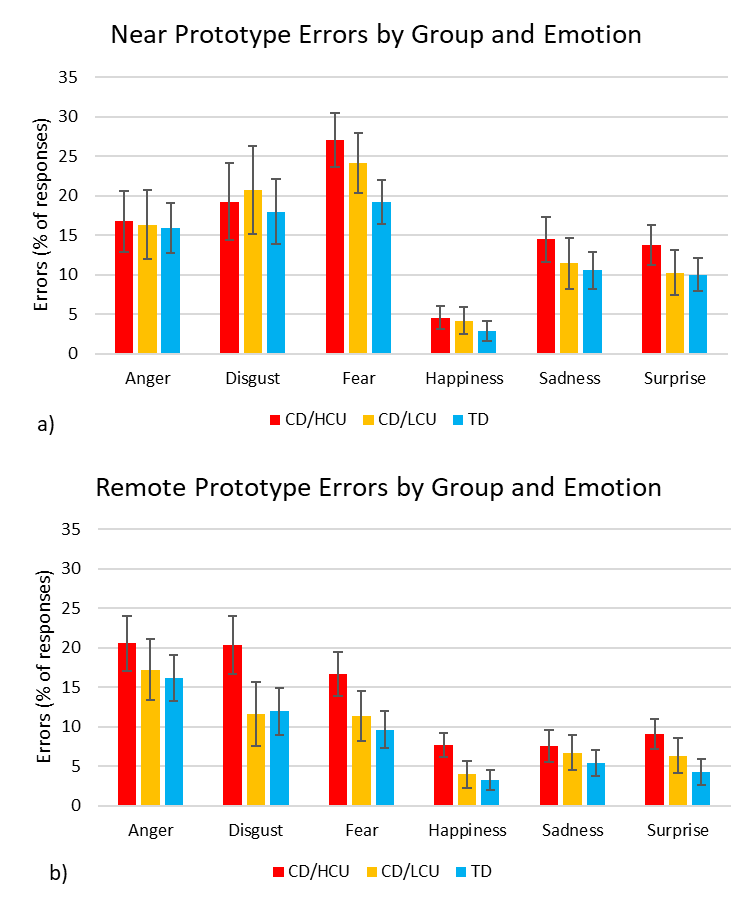


**Figure S2.** Remote and near prototype errors as a percentage of all responses by group and emotion. Error bars are 95% confidence intervals of the mean. Omnibus tests did not reveal any significant differences between group, emotion, and error type.

## SVM Classifiers

We repeated the main Angle-GMLVQ analyses using SVM classifiers, to check that performance was comparable with both methods. SVM classifiers were trained and tested using the standard MATLAB functions *fitcsvm* and *predict*. We trained and tested six classifiers for each model: linear SVMs and SVMs with second, third, fourth, fifth and sixth-order non-linear polynomial transformation kernels. For each model (CD-TD, HCU-TD, LCU-TD, and HCU-LCU), we then selected the SVM with the highest weighted accuracy for comparison with the corresponding Angle-GMLVQ classifier. The training and testing procedure for the SVM classifiers was identical to the Angle-GMLVQ classifiers, except for the classifier itself.

For each model, the linear SVM achieved the highest weighted accuracy, and thus the linear classifiers were selected for comparison with the Angle-GMLVQ models. Weighted accuracies for each SVM model and the corresponding Angle-GMLVQ models are shown in Figure S3 below. In short, performance was very similar for the Angle-GMLVQ and SVM models. The Angle-GMLVQ classifier demonstrated a small but significant advantage for the CD-TD and HCU-TD models, while the SVM classifier demonstrated a small but significant advantage for the LCU-TD and HCU-LCU models (Mann-Whitney U tests, all *p* < .05).


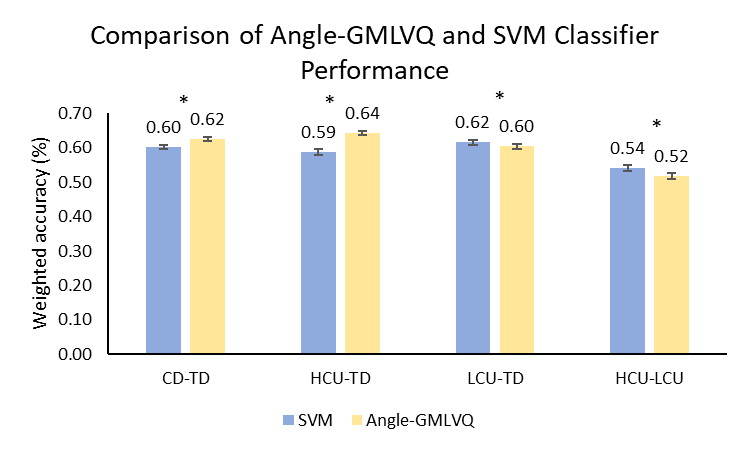


**Figure S3.** Weighted accuracies for each of the four linear SVM classifier models displayed alongside the equivalent Angle-GMLVQ models for comparison. * indicates a significant group difference (Mann-Whitney U tests, p < .05)

***Angle-GMLVQ analyses with 23 features***

The Angle-GMLVQ classification analyses were repeated using 23 features instead of 12 features, capturing the percentage of times that each emotion at each intensity level was mislabelled as each of the other emotions. Classifier performance for these analyses is shown in Table S2., below. Model performance was not generally improved by the use of this larger feature set. Interestingly, however, these models achieved higher TPRs and lower TNRs compared to the main models with 12 features, suggesting that while overall performance was similar, the use of the larger feature set increased the number of cases classified correctly (CD/HCU or CD) while reducing the number of controls detected (TD).

**Table S2.** Angle-GMLVQ model performance with 23 features (mean (95% confidence intervals of the mean))

|  | CD-TD | HCU-TD | LCU-TD | F (p), η^2^_p_ | HCU-LCU |
| --- | --- | --- | --- | --- | --- |
| Accuracy (macro-averaged) | 0.60 (0.59, 0.60) ^a^ | 0.59 (0.59, 0.60) ^a^ | 0.62 (0.61, 0.63) ^b^ | 15.21 (<.001), .09 | 0.53 (0.52, 0.54) |
| PPV | 0.69 (0.69, 0.70) ^a^ | 0.82 (0.82, 0.83) ^b^ | 0.82 (0.82, 0.83) ^b^ | 1,451.60 (<.001), .91 | 0.55 (0.54, 0.56) |
| NPV | 0.52 (0.52, 0.53) ^a^ | 0.33 (0.32, 0.34) ^b^ | 0.39 (0.38, 0.39) ^c^ | 523.97 (<.001), .78 | 0.51 (0.50, 0.52) |
| TPR | 0.75 (0.75, 0.76) ^a^ | 0.72 (0.71, 0.73) ^b^ | 0.76 (0.75, 0.77) ^a^ | 28.59 (<.001), .16 | 0.45 (0.44, 0.46) |
| TNR | 0.44 (0.44, 0.45) ^a^ | 0.47 (0.45, 0.49) ^b^ | 0.48 (0.47, 0.50) ^b^ | 7.26 (<.001), .05 | 0.61 (0.60, 0.63) |

^Notes: CD-TD, conduct disorder – typically developing model. HCU-TD, high callous-unemotional – typically developing model. LCU-TD, low callous-unemotional – typically developing model. PPV, positive predictive value. NPV, negative predictive value. TPR, true positive rate. TNR, true negative rate. η2p, partial eta squared. Groups with different superscript indices differ significantly in post-hoc comparisons (^*^p^*^<.05, Bonferroni corrected). Note that the HCU-LCU model (column 6) was not included in statistical tests as comparisons between this and other models were not relevant to hypotheses^

## Site of Data Collection

The number of participants per group recruited from each site is displayed in Table S3.

**Table S3.** Number of participants recruited from each site per group

| Site | CD/HCU  (*n* = 248) | CD/LCU  (*n* = 230) | TD  (*n* = 785) | Total  (*n* = 1263) |
| --- | --- | --- | --- | --- |
| Frankfurt | 34 | 51 | 147 | 232 |
| Aachen | 60 | 38 | 148 | 246 |
| Amsterdam | 34 | 33 | 87 | 154 |
| Southampton | 12 | 33 | 95 | 140 |
| Basel | 6 | 9 | 33 | 48 |
| Birmingham | 27 | 24 | 115 | 166 |
| Dublin | 0 | 0 | 5 | 5 |
| Barcelona | 10 | 6 | 15 | 31 |
| Bilbao | 23 | 12 | 57 | 92 |
| Budapest | 14 | 9 | 17 | 40 |
| Athens | 28 | 15 | 66 | 109 |

^Notes: CD/HCU, conduct disorder with high levels of callous-unemotional traits, CD/LCU, conduct disorder with low levels of callous-unemotional traits, TD, typically developing^

## Supplementary References

Eekhout, I., de Vet, H. C., Twisk, J. W., Brand, J. P., de Boer, M. R., & Heymans, M. W. (2014). Missing data in a multi-item instrument were best handled by multiple imputation at the item score level. *Journal of Clinical Epidemiology, 67*(3), 335-342. doi: https://doi.org/10.1016/j.jclinepi.2013.09.009

Liu, Y., & De, A. (2015). Multiple imputation by fully conditional specification for dealing with missing data in a large epidemiologic study. *International Journal of Statistics in Medical Research, 4*(3), 287. doi: 10.6000/1929-6029.2015.04.03.7
